# Real-world Impact of Integrating Comprehensive Geriatric Assessment into Clinical Treatment Decision-making for Older Patients with Bladder Cancer

**DOI:** 10.1016/j.euros.2026.07.001

**Published:** 2026-07-23

**Authors:** V.C. Rutten, N.M.A. van Rijen, M. Franckena, D.G.J. Robbrecht, T.C.M. Zuiverloon, J.L. Boormans, H.A. Polinder-Bos

**Affiliations:** a Department of Urology, Erasmus MC Cancer Institute, Rotterdam, The Netherlands; b Division of Geriatric Medicine, Department of Internal Medicine, Erasmus MC University Medical Centre, Rotterdam, The Netherlands; c Department of Radiation Oncology, Erasmus MC Cancer Institute, Rotterdam, The Netherlands; d Department of Medical Oncology, Erasmus MC Cancer Institute, Rotterdam, The Netherlands

**Keywords:** Bladder cancer, CGA, Frailty, Treatment selection, Treatment recommendations

## Abstract

**Background and objective:**

Treatment decision-making in older patients with bladder cancer (BC) is complicated by frailty and comorbidities. Comprehensive geriatric assessment (CGA) may support this process. This study evaluated the impact of CGA on treatment selection and identified CGA-prompted interventions in older patients with high-risk non–muscle-invasive BC (NMIBC) or muscle-invasive BC (MIBC).

**Design, setting, participants and intervention:**

Patients aged ≥70 yr or aged <70 yr and deemed frail were referred for CGA after multidisciplinary team (MDT) discussion. Outcomes were discussed at a subsequent MDT meeting, and changes in treatment plans were recorded. The impact of CGA was defined as (1) modification of proposed treatment, (2) assessment of functional status because of concerns about resilience for treatment, or (3) guidance among multiple treatment options. Medical records were examined for CGA-prompted interventions. Overall survival (OS) was described according to CGA impact.

**Results and limitations:**

Among 201 patients (79% male, median age 76 yr), CGA influenced treatment selection in 19% (*n* = 38). In the impact group, CGA resulted in treatment de-escalation in 25 of the 38, assessed resilience in 7 of the 38, and guided selection among multiple options in 6 of the 38 patients. A total of 443 patient-specific interventions were generated, especially medication adjustments (22%), paramedical consultations (20%), and monitoring recommendations (18%). Compared with the no-impact group, patients in the impact group had poorer physical and cognitive performance and received best supportive care more frequently. Observed median OS was 8.4 versus 40.3 mo in MIBC and 19.4 versus not reached in NMIBC, likely reflecting underlying frailty and treatment selection.

**Conclusions:**

CGA contributed to treatment selection in 1 in 5 patients, leading to de-escalation in frailer patients and supporting decisions when resilience or treatment choice was uncertain, demonstrating its potential to optimise treatment strategies.

## Take Home Message

Comprehensive geriatric assessment (CGA) contributes to treatment selection in 19% of the older patients with bladder cancer. It prompted treatment de-escalation in patients with frailty, supported decision-making by assessing patients’ functional status when concerns about resilience for treatment arose, and guided selection among multiple treatment options. These findings suggest that CGA may be a useful tool in optimising treatment strategies for older patients with bladder cancer.

ADVANCING PRACTICE **What does this study add?**

Comprehensive geriatric assessment (CGA) influenced treatment selection in 19% of the patients. When it prompted adjustments to the multidisciplinary team (MDT)–recommended treatment, these changes primarily involved treatment de-escalation. Additionally, CGA supported the assessment of patient resilience and guided the selection among multiple treatment options. Patients with CGA-driven treatment changes were frailer at baseline, received more best supportive care, and therefore had poorer observed survival. CGA facilitated protocol-driven and patient-specific interventions, including shared decision-making, paramedical consultations, medication review, and postoperative monitoring. These findings highlight that CGA has the potential to contribute to the optimisation of treatment strategies for older adults with bladder cancer.

**Clinical Relevance**

Treatment decisions for older patients with bladder cancer are frequently complicated by frailty, comorbidity, and competing health risks. This study demonstrates that integrating comprehensive geriatric assessment into multidisciplinary care can meaningfully influence treatment recommendations, identify patients who may benefit from treatment de-escalation, and uncover modifiable factors that may improve treatment tolerance and overall care delivery. These findings support broader implementation of CGA as part of routine decision-making for older patients with bladder cancer.

**Patient Summary**

This report examined how comprehensive geriatric assessment (CGA) influences treatment decision-making for older patients with bladder cancer (BC). CGA affected treatment selection in 19% of the patients by prompting less invasive treatment, providing insight into the patient’s ability to tolerate the proposed treatment or guiding choices when multiple options were available. It also yielded numerous recommendations to optimise treatment, including medication adjustments. Overall, these findings show that CGA helps to tailor and optimise treatment plans for older patients with BC.

## 1 Introduction

Bladder cancer (BC), classified as non–muscle-invasive BC (NMIBC) or muscle-invasive BC (MIBC), is the 10th most common cancer worldwide, with incidence rising with age [1]. As the population ages, the number of older patients with BC is expected to increase [2]. Management of non-metastatic MIBC includes radical cystectomy (RC) with pelvic lymph node dissection or bladder-sparing trimodality therapy (ie, transurethral resection of the bladder tumour [TURBT] followed by chemoradiotherapy [CRT]) [3]. Selected patients with high-risk NMIBC (HR-NMIBC) who experience recurrence after intravesical instillations may also opt for RC [3].

Despite advances in surgical techniques and perioperative management, RC remains a complex procedure associated with postoperative morbidity and mortality [4]. Patients aged ≥70 yr have inferior outcomes, with lower survival and higher perioperative mortality, making the risk-benefit ratio less favourable and potentially compromising quality of life (QoL) and functional outcomes [4]. CRT, in contrast, is generally well tolerated in older patients, with toxicity profiles similar to those in younger populations 5, 6, 7. These considerations highlight the importance of careful, individualised risk-benefit assessment when selecting treatment strategies for older patients.

The European Association of Urology recommends frailty assessment in older patients with MIBC [3]. Comprehensive geriatric assessment (CGA) evaluates physical, cognitive, functional, and social domains, identifying conditions that may contribute to frailty and guiding treatment decisions [8]. A systematic review of 22 studies reported that CGA influenced oncologic management in 31% of the patients, leading to treatment modifications, reduced toxicity, fewer complications, and improved physical functioning and QoL [9]. In a cohort of patients with MIBC (*n* = 86), CGA prompted treatment de-escalation in 20% of the cases, although criteria for de-escalation were not clearly defined [10]. The broader potential of CGA to direct additional interventions for optimising treatment in BC remains largely unexplored.

To address this gap and assess real-world implementation, we evaluated the impact of integrating CGA into treatment decision-making for older patients with BC. Specifically, we examined how frequently CGA influenced treatment selection in a large cohort of patients with HR-NMIBC or MIBC and identified CGA-prompted interventions aimed at optimising outcomes. Overall survival (OS) was described for patients whose treatment was modified based on CGA (impact group) versus those whose treatment remained unchanged (no-impact group).

## 2 Materials and methods

### 2.1 Study design

A single-centre, prospective observational cohort study was conducted at the Geriatrics department and Bladder Cancer Centre of Erasmus Medical Centre, Rotterdam, where patients receive counselling from a multidisciplinary team (MDT) of urologists, oncologists, and radiation oncologists. Eligible patients were those with HR-NMIBC or MIBC who were potentially eligible for RC, aged ≥70 yr, or younger but deemed at risk of frailty based on the clinical judgement of the treating physician, and who underwent CGA between June 2020 and November 2024. Patients who did not provide consent or who had concurrent upper tract tumours were excluded. A subset of this cohort has been previously published, focusing on health outcome priorities [11]. The study was approved by the Erasmus Medical Centre Medical Ethical Committee (2019-0711).

### 2.2 CGA and health-related QoL

A standardised CGA was performed by a geriatrician, a resident or a nurse practitioner specialised in geriatric medicine at the outpatient department. CGA included the following: (1) an interview including a review of medical history, medication use and sociodemographic status; (2) a physical examination including height, weight, blood pressure, including orthostatic challenge, hand grip strength, gait speed, Five Times Sit-to-Stand (FTSS) and/or Timed Up and Go Test (TUGT) measurements 12, 13, 14, 15, 16; and (3) assessments of functional, cognitive and nutritional status using Katz Activities of Daily Living (ADL) scale, Lawton Instrumental ADL (iADL) scale, Mini Nutritional Assessment Short Form, Mini-Mental State Examination (MMSE), clock drawing test, Clinical Frailty Scale (CFS) and Geriatric Depression Scale-2 17, 18, 19, 20, 21, 22, 23. The level of education was categorised into five levels according to the International Standard Classification of Education [24].

Medical history was obtained through patient interviews and reviewed in medical records. Comorbidities were recorded as present (yes) or absent (no). Definitions for comorbidities and cut-off scores for CGA determinants are provided in Supplementary Table 1.

Recent TURBT may have caused (temporary) incontinence, potentially overestimating ADL dependency. To account for this, a modified Katz score excluding continence was calculated. During the study, the CGA protocol was updated, replacing the TUGT with the FTSS. For patients without FTSS data, TUGT results were reported (FTSS: *n* = 133; TUGT: *n* = 57; both missing: *n* = 11).

At the first consultation, patients completed the EuroQol 5-Dimension (EQ-5D) and European Organisation for Research and Treatment of Cancer Quality of Life Questionnaire Core 30 (QLQ-C30) questionnaires 25, 26. QoL was assessed using the final EQ-5D question and two QLQ-C30 items, which capture patients’ perceived health status and overall QoL. Scores were categorised as poor (1–3), fair (4–5) or good (6–7).

### 2.3 Impact of CGA: treatment selection

The primary outcome was the impact of CGA on treatment selection. Patients were referred for CGA after MDT discussion, and CGA findings were presented by a geriatrician at a follow-up MDT meeting, where final treatment decisions were made.

CGA was considered to have an impact if it (1) modified the initially proposed treatment, (2) informed decisions by assessing the patient’s functional status when there were concerns about the patient’s resilience to the proposed treatment, or (3) guided selection among multiple treatment options. It was classified as having no impact if it (1) confirmed the pre-existing treatment plan, (2) treatment had already been determined or initiated, (3) the patient died before treatment, or (4) changes were because of comorbidities.

CGA impact was prospectively recorded during MDT meetings as either “CGA changed” or “CGA did not change” treatment selection. Undocumented cases were re-evaluated (N.v.R.) and discussed with a geriatrician (H.P.B.). Among patients whose treatment was influenced by CGA (impact group), patient records and MDT notes were retrospectively reviewed to assess how CGA influenced treatment selection. Treatment modification towards a less invasive approach was classified as “treatment de-escalation” (eg, RC to chemohyperthermia or CRT to best supportive care). Cases in which CGA addressed patients’ functional status because of concerns or guided selection among multiple options were categorised according to the definitions described above. When both a treatment proposal and performance concerns were present, categorisation followed the final treatment decision.

CGA’s impact on treatment selection was also analysed by year to explore differences over time.

### 2.4 Impact of CGA: interventions

Patient records were retrospectively reviewed to identify predefined CGA-prompted interventions, classified as protocol-based or patient specific. Protocol-based interventions included shared decision-making, fall and delirium prevention, and in-hospital geriatric comanagement. Patient-specific interventions included lifestyle or prehabilitation advice (eg, smoking cessation and increased activity), medication changes (eg, initiation, discontinuation, or avoidance of drugs such as nonsteroidal anti-inflammatory drugs or haloperidol), guidance on anticipated complications (eg, delirium management), referrals to medical specialists, recommendations for the general practitioner (eg, laboratory tests and blood pressure monitoring) and paramedical consultations in hospital or at home (physiotherapy, dietetics, psychology).

### 2.5 Statistical analysis

Continuous data are presented as mean ± standard deviations or medians with interquartile ranges (IQRs) for skewed distributions, and categorical variables were presented as counts with percentages. Group differences by CGA impact were assessed using the chi-square or Fisher exact test for categorical variables and the independent samples *t* test and Mann-Whitney U test for continuous variables. Chi-square tests evaluated differences in CGA impact across consultation years.

Median follow-up time was estimated using the reverse Kaplan-Meier method. OS was defined as the time from CGA to death from any cause or last follow-up (censored) and estimated using the Kaplan-Meier method. Survival curves were stratified by CGA impact (ie, “impact” vs “no impact”). Survival status was last verified on July 24, 2025. Data on the cause of death was unavailable.

A *p* value <0.05 was considered statistically significant. All analyses were performed using SPSS version 28.0.1.0 (IBM, Armonk, USA).

## 3 Results

### 3.1 Enrolment and patient characteristics

Between June 2020 and November 2024, 214 patients with MIBC or HR-NMIBC received CGA. After excluding 8 patients who declined consent and 5 with concurrent upper tract tumours, 201 patients were included. Median age was 76 yr (IQR 72–79), and 79% were male. Twenty-six patients were diagnosed with HR-NMIBC (Table 1). Prevalent comorbidities included hypertension (55%), kidney disease (49%) and prior malignancy (27%). Most patients presented with cT2–cT3 disease, with 42% exhibiting concomitant carcinoma in situ. Most patients (36%) underwent RC.

**Table 1:** Baseline and treatment characteristics of patients aged >70 yr or at risk for frailty with high-risk NMIBC and MIBC undergoing a CGA (*n* = 201), stratified for CGA impact (“impact” vs “no impact” group)

***Characteristic***	***n (%) or median (IQR)***^a^	
	**No impact (*n*** = **163)**	**Impact (*n*** = **38)**
**Age (yr)**	75 (72–79)	78 (74–81)
**Sex**		
Male	126 (77)	32 (84)
**Smoking**		
Yes	26 (16)	9 (24)
Ex-smoker	100 (61)	21 (55)
No	37 (23)	8 (21)
**Medication count**	4 (2–7)	5 (3–8)
**Level of education**		
Early childhood and primary	30 (21)	7 (23)
Lower secondary	37 (26)	5 (16)
Upper secondary	49 (35)	12 (39)
Postsecondary, nontertiary, and short cycle tertiary	19 (14)	2 (6.5)
Bachelor’s, master’s, or doctoral	6 (4.3)	5 (16)
**Housing situation**		
With partner	116 (71)	24 (63)
Alone	47 (29)	14 (37)
**BMI(kg/m^2^)**	26 (24–29)	27 (24–29)
**CCI score**	6 (5–7)	7 (6–9)
**Comorbidity**		
Previous malignancy	47 (29)	8 (21)
Myocardial infarction	31 (19)	8 (21)
Heart failure	6 (3.7)	5 (13)
Vascular disease	26 (16)	10 (26)
Hypertension	89 (55)	22 (58)
Diabetes mellitus	36 (22)	14 (37)
Pulmonary disease	25 (15)	3 (7.9)
Kidney disease	75 (46)	23 (61)
Cerebral vascular accident	21 (13)	8 (21)
Musculoskeletal problems	40 (25)	10 (26)
Psychiatric disorders	8 (4.9)	6 (16)
**Tumour type**		
Urothelial cell carcinoma	153 (94)	37 (97)
Squamous cell carcinoma	6 (3.7)	0 (0)
Other	4 (2.5)	1 (2.6)
**Concomitant CIS**	70 (44)	12 (33)
**Tumour stage**		
Ta or Tis	7 (4.3)	2 (5.3)
T1	12 (7.4)	5 (13)
T2	79 (49)	16 (42)
T3	59 (36)	13 (34)
T4	6 (3.7)	2 (5.3)
**Treatment**		
RC	70 (43)	3 (7.9)
Preoperative chemotherapy + RC^b^	13 (8.0)	0 (0)
Chemoradiotherapy	20 (12)	2 (5.3)
Radiotherapy only	21 (13)	6 (16)
Palliative chemotherapy or radiotherapy	14 (8.6)	6 (16)
Best supportive care	6 (3.7)	15 (40)
Salvage RC after CRT	6 (3.7)	0 (0)
Other^c^	13 (8.0)	6 (16)

NMIBC = non–muscle-invasive bladder cancer; MIBC = muscle-invasive bladder cancer; CGA = comprehensive geriatric assessment; SD = standard deviation; IQR = interquartile range; BMI = body mass index; CCI score = Charlson Comorbidity Index Score; CIS = carcinoma in situ; RC = radical cystectomy; CRT = chemoradiation; TURBT = transurethral resection of bladder tumour.

Data incomplete for level of education (*n* = 29), BMI (*n* = 4), and concomitant CIS (*n* = 7).

a Data displayed as *n* (%) or median [IQR]. Column percentages per variable may not add up to 100% because of rounding.

b Preoperative chemotherapy is either neoadjuvant or induction chemotherapy.

c Includes: induction chemotherapy without RC because of disease progression (*n* = 4), death before treatment (*n* = 5), Bricker ileal conduit without RC (*n* = 1), TURBT and/or intravesical chemo hyperthermia (*n* = 7) and lymph node dissection (LND) only because of *N*+ disease during LND (*n* = 2).

Assessment of physical and cognitive functioning (Table 2) showed that 31% were iADL dependent. After excluding continence from the Katz Index, 8% remained ADL dependent. Malnutrition or risk thereof was present in 34%, and 64% were not frail (CFS 1–3).

**Table 2:** Determinants of physical and cognitive functioning and quality of life questionnaires of patients aged >70 yr with high-risk NMIBC and MIBC undergoing a CGA (*n* = 201), stratified for CGA impact (“impact” vs “no impact” group)

***Determinant***	***n (%), mean*** ± ***SD or median (IQR)***^a^		
	**No impact (*n*** = **163)**	**Impact (*n*** = **38)**	***p* value**
**Lawton score**	0 (0–3)	5 (0–9)	<0.001
iADL dependent (≥4)	39 (24)	22 (58)	
**Katz score**	0 (0–1)	1 (0–1)	0.002
ADL dependent (≥1)	56 (35)	23 (62)	
**Katz score excluding incontinence**	0 (0–0)	0 (0–0)	0.007
ADL dependent (≥1)	9 (5.6)	7 (19)	
**Use of a walking aid**	26 (16)	10 (26)	0.13
**Falls in the previous 12 mo**	33 (21)	11 (29)	0.3
**Orthostatic hypotension present**	40 (33)	10 (39)	0.6
**MNA-SF score**	13 (11–14)	11 (8–13)	0.003
(At risk of) malnutrition (≤11)	47 (29)	20 (57)	
**Clock test score**	13 (12–14)	12 (10–13)	0.003
Impaired (<11)	20 (14)	9 (29)	
**MMSE score**	28 (27–29)	27 (24–29)	<0.001
Impaired (<24)	5 (3.2)	7 (19)	
**Clinical Frailty Scale score**			<0.001
1–3	114 (73)	8 (24)	
4–5	39 (25)	22 (65)	
>6	3 (1.9)	4 (12)	
**GDS-2, depressive symptoms present**	8 (7.4)	6 (24)	0.02
**Grip strength (kg)**			
Male	36 ± 7.7	32 ± 8.9	0.01
Female	24 ± 3.6	21 ± 2.6	0.03
Low grip strength	19 (12)	10 (26)	
**Gait speed (m/s)**	1.2 ± 0.27	0.93 ± 0.20	<0.001
Low gait speed	20 (13)	12 (34)	
**Five Times Sit to Stand Test (s)**	10.4 (9.1–13.7)	14.1 (11.2–17.9)	<0.001
**Timed Up and Go Test (s)**	9.0 (7.5–10.0)	11.1 (11.0–13.0)	0.01
**EQ5D score** How would you rate your health today on a scale from 0–100?	76 (60–86)	78 (47–80)	0.4
**EORTC QLQ-C30 score** How would you rate your overall health during the past week?	5 (5–6)	5 (4–6)	0.5
Poor	15 (13)	3 (14)	
Fair	45 (38)	11 (50)	
Good	59 (50)	8 (36)	
How would you rate your overall quality of life during the past week?	6 (5–6)	5 (4–6)	0.5
Poor	16 (13)	3 (13)	
Fair	43 (36)	11 (48)	
Good	60 (50)	9 (39)	

NMIBC = non–muscle-invasive bladder cancer; MIBC = muscle-invasive bladder cancer; CGA = comprehensive geriatric assessment; SD = standard deviation; IQR = interquartile range; iADL = instrumental activities of daily living; ADL = activities of daily living; MNA-SF = Mini Nutritional Assessment Short Form; MMSE = Mini-Mental State Examination; GDS = Geriatric Depression Scale; EQ5D = EuroQol 5D; EORTC QLQ-C30 = European Organisation for Research and Treatment for Cancer Quality of Life Questionnaire.

Data incomplete for falls last year (*n* = 2), orthostatic hypotension (*n* = 54), MNA-SF (*n* = 6), clock test (*n* = 23), MMSE (*n* = 7), Clinical Frailty Scale (*n* = 11), GDS-2 (*n* = 68), grip strength men (*n* = 1), gait speed (*n* = 6), Five Times Sit to Stand Test (*n* = 76), Timed Up and Go Test (*n* = 144), EORTC QLQ-C30 overall health (*n* = 59), EORTC QLQ-C30 quality of life (*n* = 60), and EQ5D (*n* = 86).

a Data displayed as *n* (%), median [IQR], or mean ± SD. Column percentages per variable may not add up to 100% because of rounding.

### 3.2 Impact of CGA: Treatment selection

In 163 patients (81%), MDT treatment decisions remained unchanged following CGA (no-impact group). In 155 of the 163 patients (95%), CGA supported the intended treatment. In 8 of the 163 patients (5%), treatment remained unchanged because it was already determined (*n* = 1) or initiated (*n* = 3), the patient had died (*n* = 1), or changes were made because of comorbidities (*n* = 2). For 2 patients, documentation was insufficient to assess CGA impact, and they were classified as no impact.

CGA influenced treatment selection in 38 patients (impact group, 19%). In 25 of the 38 patients (66%), CGA prompted modification of the initial treatment plan, resulting in treatment de-escalation. In 7 of the 38 patients (18%), CGA informed treatment decisions by addressing concerns about patients’ resilience to the proposed treatment. In 6 of the 38 patients (16%), CGA guided selection among multiple treatment options. Proposed and final treatments for the impact group are summarised in Supplementary Table 2.

The proportion of patients in whom CGA influenced treatment selection differed across years (Supplementary Table 3), peaking in 2023 (30%) and 2024 (32%), despite most referrals occurring in 2021 and 2022 (c^2^[4] = 9.45, *p* = 0.05).

Patients in the impact group more frequently had heart failure (13% vs 3.7%) and diabetes (37% vs 22%) and were more likely to receive best supportive care (40% vs 3.7%) compared with those in the no-impact group, who mostly received curative treatment (RC 43%; CRT: 12%; Table 1). The impact group also had poorer functional and cognitive performance, with higher iADL and ADL dependency, greater malnutrition risk, lower MMSE and clock test scores, higher CFS scores, reduced grip strength, slower gait speed and more frequent FTST and TUGT impairments (Table 2).

### 3.3 Impact of CGA: Interventions and actions

A total of 481 protocol-based interventions were delivered to 201 patients, with all receiving shared decision-making. Geriatric comanagement during hospitalisation was recommended for 61%, and 79% received guidance on fall and delirium prevention. Additionally, CGA provided 443 patient-specific interventions to 175 patients. Twenty-six patients (13%) did not receive any patient-specific intervention. The most common interventions included medication changes (22%), in-hospital paramedical consultations (20%) and postoperative monitoring recommendations (18%; Table 3).

**Table 3:** CGA-prompted interventions and actions in patients aged >70 yr with high-risk NMIBC or MIBC (*n* = 201)

***Intervention and action***	***Number of recommendations given***	
**Protocol based (yes/no)**^a^	***n* (%)**	
Shared decision making	201 (100)	
Geriatric comanagement during hospitalisation	122 (61)	
Fall and delirium prevention strategies	158 (79)	
**Total**	481	
***Intervention and action***	***Total number of recommendations given per category***	***Number of patients receiving this recommendation***

**Patient-specific (number)**^b^	***n* (%)**	***n* (range per patient)**

Lifestyle and prehabilitation advice	58 (13)	43 (1–3)
Medication changes	96 (22)	71 (1–3)
Consultations with other medical specialists	24 (5.4)	24 (1–1)
Recommendations for postoperative in-hospital monitoring	80 (18)	70 (1-4)
Recommendations for a general practitioner	64 (14)	39 (1–3)
Paramedics: consultation during hospitalisation	89 (20)	74 (1–2)
Paramedics: consultation at home	32 (7.2)	26 (1–3)
**Total**	443 (99^c^)^c^	347

CGA = comprehensive geriatric assessment; NMIBC = non–muscle-invasive bladder cancer; MIBC = muscle-invasive bladder cancer; n.a. = not applicable.

a Only one recommendation given per patient; scored as yes/no.

b Multiple recommendations given per patient.

c Does not add up to 100 because of rounding.

### 3.4 OS

**Fig. 1:**
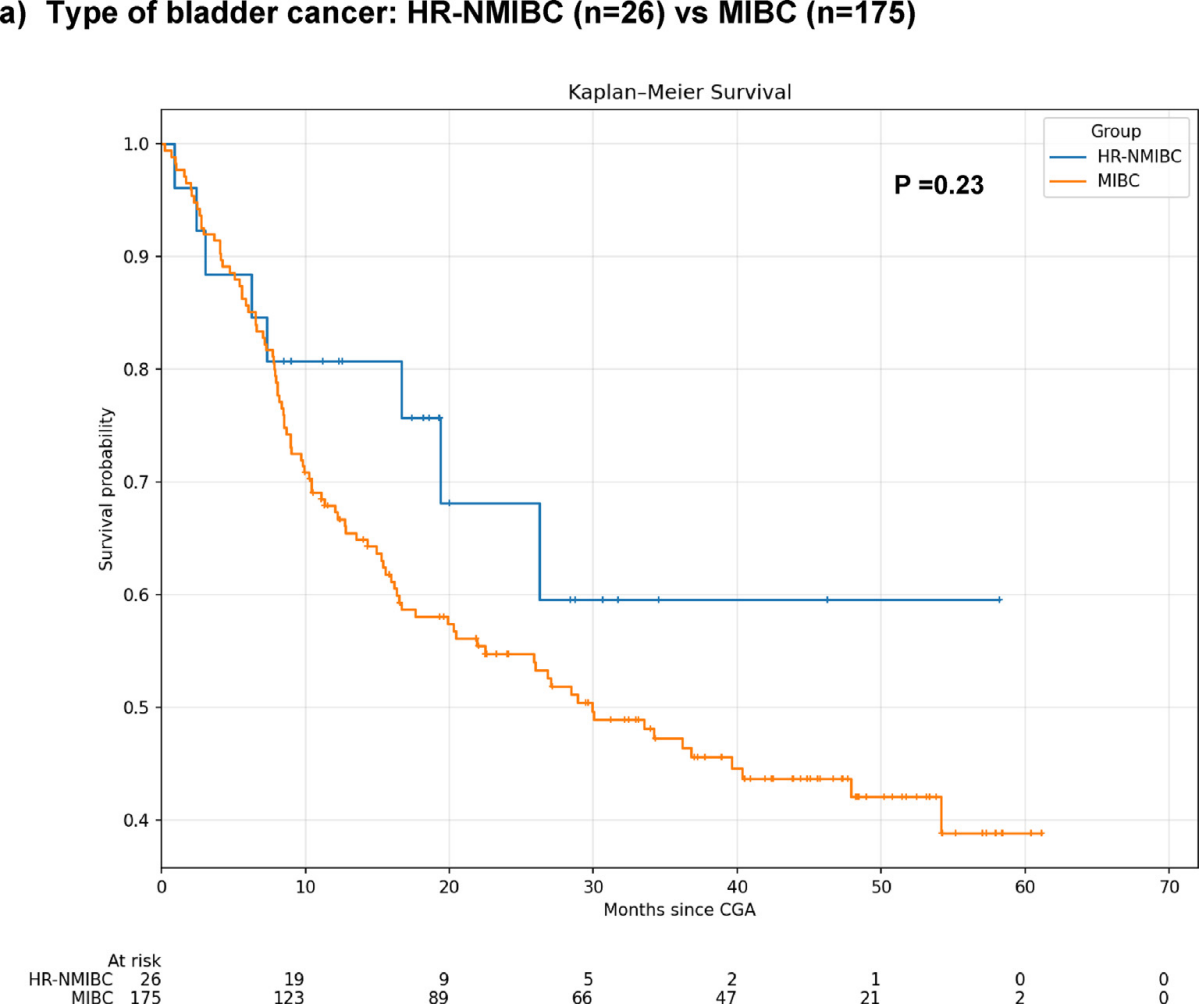

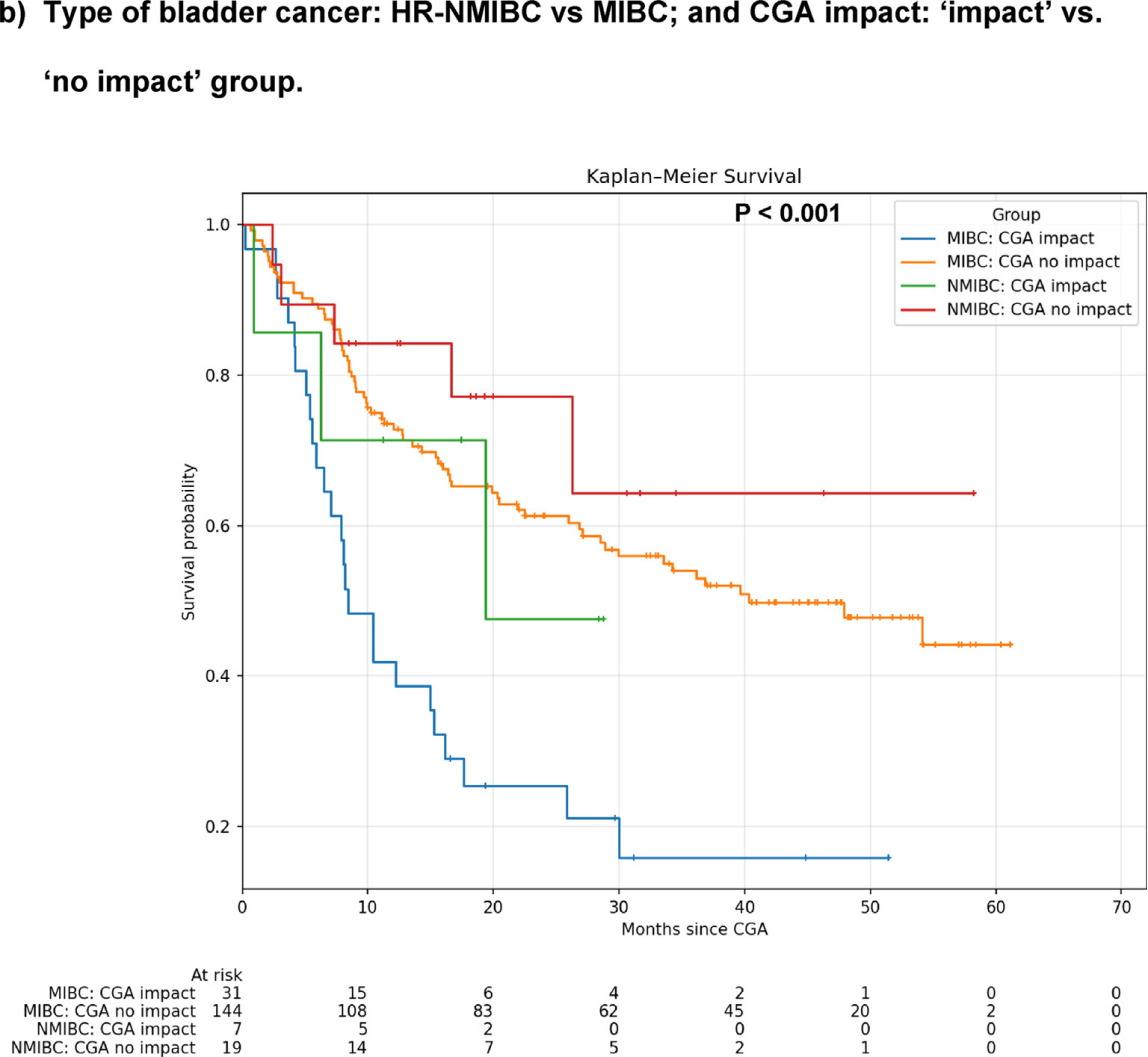
Kaplan-Meier survival curve demonstrating the survival probability for patients aged >70 yr with HR NMIBC and MIBC undergoing a CGA (*n* = 201) stratified by (A) type of bladder cancer, that is, HR-NMIBC (*n* = 26) versus MIBC (*n* = 175) and (B) type of bladder cancer, that is, HR-NMIBC versus MIBC and CGA, that is, “impact” versus “no impact” groups. HR = high risk; NMIBC = non–muscle-invasive bladder cancer; MIBC = muscle-invasive bladder cancer; CGA = comprehensive geriatric assessment.

During a median follow-up of 42 mo (95% CI 36–48), 101 deaths were observed. Median OS was not reached for HR-NMIBC, whereas patients with MIBC had a median OS of 30 mo (95% CI 17–43; Fig. 1A).

When stratified by CGA impact, OS differed between groups. In patients with MIBC, the observed median OS was 8.4 mo (95% CI 5.6–11) in the CGA impact group and 40 mo (95% CI 24–57) in the no-impact group. In patients with NMIBC, median OS was 19 mo (95% CI not estimable) in the impact group, whereas median OS was not reached in the no-impact group (Fig. 1B).

## 4 Discussion

In this cohort of older patients with HR-NMIBC and MIBC, CGA influenced treatment selection in 19% of the patients. CGA most often led to treatment de-escalation, was used to evaluate patients’ resilience, and supported decision-making when multiple treatment options were available. Patients whose treatment was modified had poorer physical and cognitive performance and were more often directed towards best supportive care. Observed survival differences between the impact and no-impact groups likely reflect underlying baseline frailty and resulting treatment decisions, rather than the effect of CGA itself. Despite greater functional impairments, QoL was similar between groups, likely reflecting its multidimensional nature. Factors such as adaptation, coping and social support also influence QoL, meaning that objective impairments do not necessarily translate into differences in self-reported QoL. CGA also generated patient-specific interventions, including shared decision-making, paramedical consultations, medication adjustments and postoperative monitoring.

These findings align with previous studies showing that CGA frequently alters oncological treatment plans in older adults. In a cohort of patients with nonmetastatic MIBC (*n* = 86, median age 83.5 yr), CGA modified treatment in 20% of the patients [10]. Studies in broader oncology populations report rates of 22–27%, usually towards less intensive or palliative care 9, 27. Patients with modified treatment were generally frailer, had more polypharmacy and more ADL/iADL limitations [27]. Together, this suggests that CGA may be a valuable tool for identifying patients who may benefit from treatment de-escalation. Although it requires additional resources, it may reduce overall costs by identifying patients with frailty at high risk of complicated clinical courses 28, 29.

Beyond treatment selection, CGA provides targeted interventions to optimise treatment. In a retrospective cohort of 500 older patients undergoing elective surgery, preoperative CGA resulted in medication changes in 75% of the patients, lifestyle advice in 54%, and therapeutic interventions in 23% [30]. Ultimately, 15% of the patients refrained from surgery; these patients were more likely to have benign pathology, higher frailty scores, and lower functional status [30]. A meta-analysis of 33 studies reported that 72% of the patients received at least one nononcologic recommendation, commonly targeting polypharmacy (38%), comorbidity optimisation (30%), and nutritional support (37%) [9]. In our cohort, fewer patients received such recommendations, likely because of extensive prescreening by three medical specialists prior to MDT discussion. Nevertheless, our findings highlight CGA’s broader value in optimising care.

A key question remains whether CGA improves clinical outcomes such as perioperative morbidity, length of stay and mortality. Evidence from randomised controlled trials in urological oncology is currently lacking, although ongoing studies may provide clarity. A Danish randomised trial (NCT05679557) is currently evaluating preoperative CGA in older patients with BC undergoing RC, focusing on outcomes including days alive and out of hospital, complications and mortality [31].

Key requirements for implementing CGA in oncological care are collaboration and commitment. Integrating a geriatrician into the MDT and tumour board meetings is essential to build relationships, support team learning and combine expertise to determine the best treatment for each patient. In our study, the proportion of cases in which CGA affected treatment selection differed across years. We assume that this reflects better identification of patients who benefit from CGA, increased confidence through ongoing collaboration among medical specialists, and insights gained from reviewing postoperative mortality in necrology meetings, which facilitated team reflection and learning.

Strengths of this study include its prospective design, large sample size, and detailed documentation of CGA. Limitations include missing data in the prospective documentation of CGA impact, particularly in earlier years. Retrospective interpretation of MDT notes may have missed nuances and clinical considerations, thereby introducing potential bias or misclassification. In 5 of the 163 patients (3.1%) classified in the no-impact group, treatment had already been initiated or the patient died prior to MDT discussion. These patients were retained to reflect real-world clinical practice, where time and logistic constraints can limit timely CGA implementation, but results should be interpreted with some caution. Assessing CGA impact solely through its effect on treatment selection may underestimate its broader influence on MDT discussions and patient counselling. Furthermore, assessment of QoL was limited because of insufficient follow-up data. Finally, patients planned for chemoradiation were less often referred for CGA than surgical candidates, leading to their underrepresentation in the cohort and potentially resulting in an underestimation of CGA’s impact in real-world clinical practice.

## 5 Conclusions

In conclusion, CGA influenced treatment selection in 19% of the cases. It prompted modifications in MDT-recommended treatment plans, most commonly resulting in de-escalation, supported decision-making by assessing functional status when concerns about treatment resilience arose, and guided selection among multiple treatment options. Patients whose treatment was modified based on CGA findings were frailer at baseline, more frequently received best supportive care, and consequently had poorer survival. These findings suggest that CGA may be a valuable tool for identifying older patients who may benefit from treatment de-escalation and for supporting personalised treatment strategies, although robust evidence evaluating its effect on clinical outcomes and quality of life is still lacking.

  ***Author contributions:*** Vera Rutten had full access to all the data in the study and takes responsibility for the integrity of the data and the accuracy of the data analysis.

  *Study concept and design:* Rutten, Boormans, Polinder-Bos.

*Acquisition of data:* Rutten, van Rijen, Zuiverloon, Boormans, Franckena, Robbrecht, Polinder-Bos.

*Analysis and interpretation of data:* Rutten, van Rijen, Polinder-Bos.

*Drafting of the manuscript:* Rutten, van Rijen.

*Critical revision of the paper for important intellectual content*: Franckena, Robbrecht, Zuiverloon, Boormans, Polinder-Bos.

*Supervision*: Zuiverloon, Boormans, Polinder-Bos.

*Other:* None*.*

  ***Financial disclosures:*** None.

  ***Funding/Support and role of the sponsors:*** None.

  ***Acknowledgements***: We would like to thank all former research students and residents of the Division of Geriatrics at the Erasmus MC University Medical Centre for their contribution to the study database.

## Appendix A Supplementary data

Supplementary Data 1 Comorbidity Definitions and Detailed Impact of CGA on Treatment Selection.
