# Supplementary material for: Real-world Impact of Integrating Comprehensive Geriatric Assessment into Clinical Treatment Decision-making for Older Patients with Bladder Cancer
Source: Eur Urol Open Sci. 2026 Jul 23;91:23–31. doi: 10.1016/j.euros.2026.07.001 (PMC13425889; doi:10.1016/j.euros.2026.07.001)
Supplement: Supplementary Data 1 — Comorbidity Definitions and Detailed Impact of CGA on Treatment Selection. [file mmc1.docx]

**Supplementary Table 1. Definitions of co-morbidities and cut-off scores for CGA determinants.**

| ***Co-morbidities*** | ***Definition*** |
| --- | --- |
| Previous malignancy | All past malignancies, apart from the currently diagnosed (or recurrence of) bladder cancer. |
| Myocardial infarction | History of a myocardial infarction or coronary artery bypass graft surgery. |
| Heart failure | History of heart failure or an ejection fraction <40%. |
| Vascular disease | History of peripheral artery disease, aneurysms, or a history of vascular interventions. |
| Pulmonary disease | History of chronic obstructive pulmonary disease, asthma or daily use of inhalers. |
| Kidney disease | History of chronic kidney disease, an estimated glomerular filtration rate <60 ml/min/1,73m^2^, kidney transplantation or nephrectomy. |
| Cerebral vascular accidents | i.e. an ischemic or haemorrhagic cerebral infarction or a transient ischemic attack. |
| Musculoskeletal problems | History of rheumatoid arthritis, total hip or knee prosthetics, severe arthrosis, spinal disc herniation, polyneuropathy or gout with preventive medication. |
| Psychiatric problems | History of diagnosed psychiatric conditions, use of antipsychotics or antidepressants, treatment in a mental health facility or a history of suicide attempts. |
| ***CGA determinants*** | ***Cut-off scores*** |
| IADL dependence | Lawton score of ≥4, considering traditional gender roles in housekeeping activities unrelated to health limitations in older men. ^1,2^ |
| ADL dependence | Katz score of ≥1.^3^ |
| Orthostatic hypotension | Decrease in systolic blood pressure of ≥20 mmHg and/or a decrease in diastolic blood pressure of ≥10 mmHg during 3 blood pressure measurements performed within 3 minutes.^4^ |
| MNA-SF score | (at risk of) malnutrition: score 0-11  Normal: score 12-14.^5^ |
| Low grip strength | <16 kg for women and <27 kg for men.^6^ |
| Low gait speed | ≤0.8 m/s.^7^ |

**References**

1. Lawton MP, Brody EM. Assessment of Older People: Self-Maintaining and Instrumental Activities of Daily Living. *The Gerontologist*. 1969;9(3):179-186.

2. Sheehan C, Tucker-Drob E. Gendered expectations distort male-female differences in instrumental activities of daily living in later adulthood. *J Gerontol B Psychol Sci Soc Sci*. 2019;74(4):715-23. doi:10.1093/geronb/gbw209

3. Katz S, Ford AB, Moskowitz RW, Jackson BA, Jaffe MW. Studies of illness in the aged. The index of ADL: a standardized measure of biological and psychosocial function. *JAMA*. 1963;185:914-9. doi:10.1001/jama.1963.03060120024016

4. Consensus statement on the definition of orthostatic hypotension, pure autonomic failure, and multiple system atrophy. The Consensus Committee of the American Autonomic Society and the American Academy of Neurology. *Neurology*. 1996;46(5):1470. doi:10.1212/wnl.46.5.1470

5. Rubenstein LZ, Harker JO, Salvà A, Guigoz Y, Vellas B. Screening for undernutrition in geriatric practice: developing the short-form mini-nutritional assessment (MNA-SF). *J Gerontol A Biol Sci Med Sci*. 2001;56(6):M366-72. doi:10.1093/gerona/56.6.m366.

6. Dodds RM, Syddall HE, Cooper R, et al. Grip strength across the life course: normative data from twelve British studies. *Plos One*. 2014;9(12):e113637. doi:10.1371/journal.pone.0113637
