# Supplementary material for: Real-world Impact of Integrating Comprehensive Geriatric Assessment into Clinical Treatment Decision-making for Older Patients with Bladder Cancer
Source: Eur Urol Open Sci. 2026 Jul 23;91:23–31. doi: 10.1016/j.euros.2026.07.001 (PMC13425889; doi:10.1016/j.euros.2026.07.001)
Supplement: Supplementary Data 2 [file mmc2.docx]

**Supplementary Table 2. Details of the proposed treatment plan prior to and after CGA assessment, for patients aged 70 years or older with high-risk NMIBC and MIBC whose CGA impacted treatment selection (n=38).**

| Case  number | Proposed treatment prior to CGA | Poor performance confirmed? (Y/N) | Additional imaging? (Y/N) | Proposed treatment after CGA | Impact category |
| --- | --- | --- | --- | --- | --- |
| #1 | RARC | n.a. | N | Chemohyperthermia | De-escalation |
| #2 | Suspicion of impaired performance, CRT | N | N | EBRT | De-escalation |
| #3 | Suspicion of impaired performance, CRT | Y | Y | BSC, EBRT if complaints | De-escalation |
| #4 | Suspicion of impaired performance, RARC | Y | N | BSC, EBRT if complaints | De-escalation |
| #5 | CRT | n.a. | N | BSC, EBRT if complaints | De-escalation |
| #6 | CRT | n.a. | N | BSC, EBRT if complaints | De-escalation |
| #7 | CRT | n.a. | N | EBRT | De-escalation |
| #8 | RARC | n.a. | N | re-TURBT | De-escalation |
| #9 | CRT | n.a. | N | BSC, EBRT if complaints | De-escalation |
| #10 | CRT | n.a. | N | BSC, EBRT if complaints | De-escalation |
| #11 | RARC | n.a. | N | No RARC, consider follow-up with cystoscopies, TURBT or EBRT if complaints | De-escalation |
| #12 | RARC | n.a. | N | EBRT | De-escalation |
| #13 | Ileal conduit + II | n.a. | N | Follow-up with cystoscopies | De-escalation |
| #14 | RARC | n.a. | N | BSC | De-escalation |
| #15 | RARC + RARNU | n.a. | N | BSC | De-escalation |
| #16 | RARNU + CRT | n.a. | N | BSC | De-escalation |
| #17 | RARC | n.a. | N | re-TURBT + II | De-escalation |
| #18 | RARC | n.a. | N | BSC | De-escalation |
| #19 | CRT or RARC | n.a. | Y | Palliative chemo | De-escalation |
| #20 | RARC or EBRT | n.a. | Y | BSC | De-escalation |
| #21 | EBRT | n.a. | Y | BSC, EBRT if complaints | De-escalation |
| #22 | RARC | n.a. | Y | EBRT | De-escalation |
| #23 | RARC or CRT | n.a. | N | EBRT | De-escalation |
| #24 | CRT | n.a. | N | BSC | De-escalation |
| #25 | RARC | n.a. | N | Palliative chemo | De-escalation |
| #26 | Suspicion of impaired performance | Y | N | Prehabilitation, reconsider RARC afterwards | Performance assessment |
| #27 | Suspicion of impaired performance | Y | N | BSC, EBRT if complaints | Performance assessment |
| #28 | Suspicion of impaired performance | Y | N | CRT | Performance assessment |
| #29 | Suspicion of impaired performance | Y | N | BSC, EBRT if complaints | Performance assessment |
| #30 | Suspicion of impaired performance | Y | Y | BSC | Performance assessment |
| #31 | Suspicion of impaired performance | Y | Y | Palliative chemo | Performance assessment |
| #32 | Suspicion of impaired performance | N | N | RARNU + CRT | Performance assessment |
| #33 | RARC or EBRT | n.a. | N | EBRT | Guide treatment selection |
| #34 | CRT or BSC | n.a. | N | BSC, EBRT if complaints | Guide treatment selection |
| #35 | RARC or BSC | n.a. | N | RARC | Guide treatment selection |
| #36 | RARC or resection bladder diverticulum or re-TURBT + II | n.a. | N | re-TURBT | Guide treatment selection |
| #37 | RARC or CRT | n.a. | N | CRT | Guide treatment selection |
| #38 | CRT or BSC | n.a. | N | BSC | Guide treatment selection |

Abbreviations: BSC = best supportive care, CRT = chemoradiation, EBRT = external beam radiotherapy, II = intravesical installations, n.a. = not applicable, RARC = robot-assisted radical cystectomy, RARNU = robot-assisted radical nefro-ureterectomy, TURBT = transurethral resection of bladder tumor.
