# Supplementary material for: Real-world Impact of Integrating Comprehensive Geriatric Assessment into Clinical Treatment Decision-making for Older Patients with Bladder Cancer
Source: Eur Urol Open Sci. 2026 Jul 23;91:23–31. doi: 10.1016/j.euros.2026.07.001 (PMC13425889; doi:10.1016/j.euros.2026.07.001)
Supplement: Supplementary Data 3 [file mmc3.docx]

**SupplementaryTable 3. Percentual impact of CGA on treatment selection per year of consultation (n=201).**

| **Year of consultation** | **Number of patients per year** | **No impact**  (n=163) | **Impact**  (n=38) | **P-value** |
| --- | --- | --- | --- | --- |
|  |  | ***n (%)*** | ***n (%)*** |  |
| 2020^a^ | 35 | 31 (89) | 4 (11) | 0.051 |
| 2021 | 53 | 47 (89) | 6 (11) |  |
| 2022 | 48 | 40 (83) | 8 (17) |  |
| 2023 | 37 | 26 (70) | 11 (30) |  |
| 2024^b^ | 27 | 19 (68) | 9 (32) |  |

^a^ Inclusion started in June 2020.
^b^ Inclusion ended after November 2024. 
Abbreviations: CGA = comprehensive geriatric assessment
